# Analyzing and comparing complex environmental time series using a cumulative sums approach

**DOI:** 10.1016/j.mex.2019.03.014

**Published:** 2019-04-09

**Authors:** Peter Regier, Henry Briceño, Joseph N. Boyer

**Affiliations:** a Center for Water and the Environment, University of New Mexico, Albuquerque, NM, United States; b Southeast Environmental Research Center, Florida International University, Miami, FL, United States; c Center for Research & Innovation, Plymouth State University, Plymouth, NH, United States

**Keywords:** Applying cumulative sums to environmental data, Cumulative sums, Driver-response, Time series, Everglades restoration, Nutrients, Water management

## Abstract

Cumulative sums (Cusums) are a simple, efficient statistical method developed for process control and increasingly used to determine underlying features of time series. Here, two useful applications of Cusums to environmental time series are presented: Cusums in the time domain and plotting Cusum-transformed variables against non-transformed variables to extract meaning in the context of driver-response relationships. These statistical analyses are simple to conduct and provide valuable information about trends, patterns and thresholds of time-series over time and in relation to potential driver variables. In addition, this work investigates the robustness of the Cusum transform to various characteristics of environmental time series that challenge conventional statistical methods. In summary, this work presents:

• Cusum methods to derive meaning from complex environmental time series.
• Effects of common time series issues on the Cusums method.
• Application to real-world datasets.

## Graphical abstract

**Figure:**
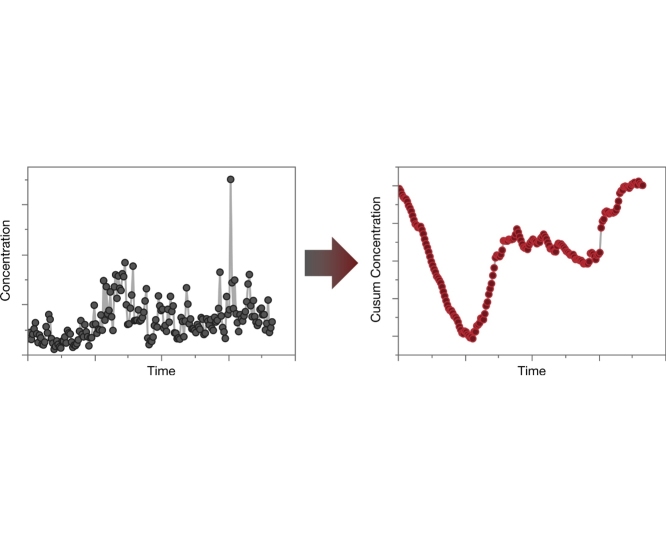

**Specifications Table**

**Table:** 

Subject Area:	*Environmental Science*
More specific subject area:	*Time series analysis*
Method name:	*Applying cumulative sums to environmental data*
Name and reference of original method:	P*age, E.S., 1954. Continuous Inspection Schemes. Biometrika 41, 100–115.* https://doi.org/10.1093/biomet/41.1-2.100
Resource availability:	*A simple Excel spreadsheet is attached as supplementary material which shows the calculation to produce Cusums presented in the manuscript*

## Method details

### Background

Proliferation of long-term data records of environmental parameters provide a valuable opportunity to adapt time series analysis techniques from other disciplines, like economics, to analyze patterns, trends, and thresholds in environmental datasets (e.g. [1]). However, environmental time series are often characterized by factors that confound standard analysis, including non-normal distributions, serial correlations, fluctuating means and variance, data gaps, outliers, and potential regime shifts within the time series ([[2], [3], [4]]). Conventional goodness-of-fit metrics like correlation/regression are not applicable to auto-correlated time series as they violate the assumption of variable independence [5]. Hence, significant data manipulation is often required prior to analysis, which slows down data interpretation and may lead to the creation of artifacts within the dataset (e.g [6]).

To overcome these obstacles, some authors have used the cumulative sums method (Cusum, [7]) to analyze time series. Cusum charts and analysis are well established in the field of industrial process control ([[8], [9], [10]]) and more recently have gained popularity in environmental sciences, including aquatic biology ([11,12]), biogeochemistry ([13,14]), climate change ([[15], [16], [17]]), and in commercial software for time series analysis ([18]).

This work 1) details a simple method to calculate and create Cusum plots, allowing users to visualize and interpret complex time series characteristics; 2) investigates the robustness of the Cusums method to overcome artifacts commonly found in environmental time series data; and 3) presents novel application of Cusums to visualize and quantify driver-response relationships. The authors see great potential for increased use of these simple techniques within the broader environmental science community, particularly insights into driver-response relationships derived from Cusums as described below.

### Cusum plots – construction and properties

Cusums are the cumulative sum of standardized deviations from a target specification, calculated as a running sum of data (z_is_) normalized to the dataset mean (m) and standard deviation (σ). To calculate Cusums, data are first standardized (Eq. (1)):

(1) z_i_ = (x_i_-m)/σ

where z_i_ is the standardized value for x_i_, the i^th^ value in the time series. Second, the cumulative sum of standardized values is calculated (Eq. (2)):

(2) z_is_ = z_i_ + z_is_-1

The distribution of resulting Cusum series has a mean = 0 and a σ = 1. Units for z_is_ are multiples of the σ. In Cusum space, positive z_is_ values indicate a deviation of x_i_ above the mean, while negative z_is_ values indicate a deviation below the mean. Decreasing and increasing slopes in Cusum trends indicate values (on average) below and above the dataset mean, respectively. Breakpoints where Cusum trends transition from negative to positive slopes (or vice versa), represent a shift in the data values from lower to higher than the dataset mean (or vice versa). To make the Cusum calculations explicit, an Excel spreadsheet is provided showing the simple calculations made to generate the Cusums presented in the figures as supplementary material.

### Visualization and interpretation of Cusum plots

**Fig. 1:**
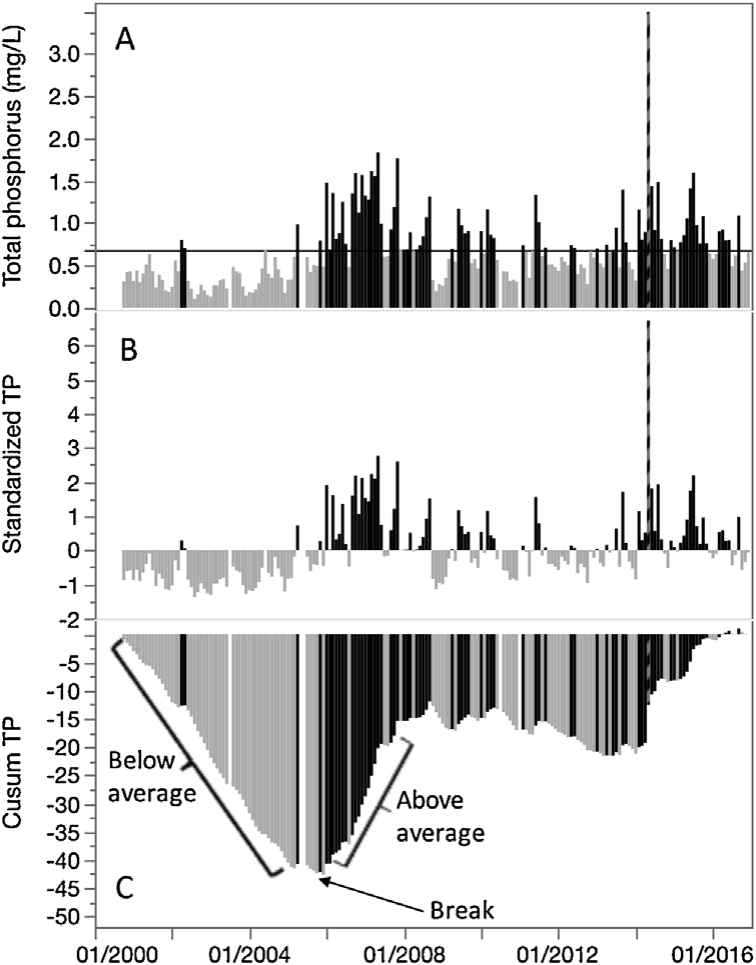
A) monthly data for total phosphorus (TP) concentrations collected at SRS-5 color-coded as above average (black) and below average (gray) values, with the dataset mean shown as a horizontal black line. B) Using Eq. (1), data are standardized by dataset mean and standard deviation. C) Using Eq. (2), cumulative sums (Cusums) of standardized data are calculated. Key features of Cusums, including increasing and decreasing slopes, and breakpoints are indicated.

In order to make examples easily reproducible, publicly available data collected by the Florida Coastal Everglades Long Term Ecological Research (FCE-LTER) project were used, which can be accessed via the project’s online database (http://fcelter.fiu.edu/data/core). Daily raw total phosphorus (TP) concentration data (dataset: LT_ND_Grahl_001 [19]) from SRS-5, located within the tidal zone of the Shark River in the Florida Everglades were binned to monthly averages and are presented as a time series in Fig. 1A with data points distinguished by color as above or below the time series mean. Clearly, there are extended, contiguous periods where TP data is higher or lower than the dataset average. Data were standardized using Eq. (1) (Fig. 1B), and then the Cusum was calculated using Eq. (2) to produce a transformed time series (Fig. 1C). Consistently negative slopes indicate periods when the majority of values are below the dataset average, while positive slopes indicate periods when the majority of values are above the dataset average.

### Statistical robustness of the Cusum approach

Along with the benefits of simple calculation, the Cusum methodology is particularly well-suited for complex ecological datasets, where conventional data analysis is often plagued by issues related to data gaps, different statistical distributions, and noise. To test the potential impact of each of these confounding factors on Cusum-transformed data, examinations of each confounding factor are presented through manipulation of the raw TP data by: 1) creating random gaps in the dataset; 2) altering the data distribution; and 3) adding white noise. Changes in Cusum characteristics based on these manipulations were quantified using the Nash-Sutcliffe model efficiency coefficient (NSE, [20]), typically used to assess goodness-of-fit of hydrologic models, where NSE = 1 indicates a perfect fit. Prior to testing, data were normalized between 0 and 1, then NSE statistics were calculated in R using the *hydroGOF* package ([21]).

**Fig. 2:**
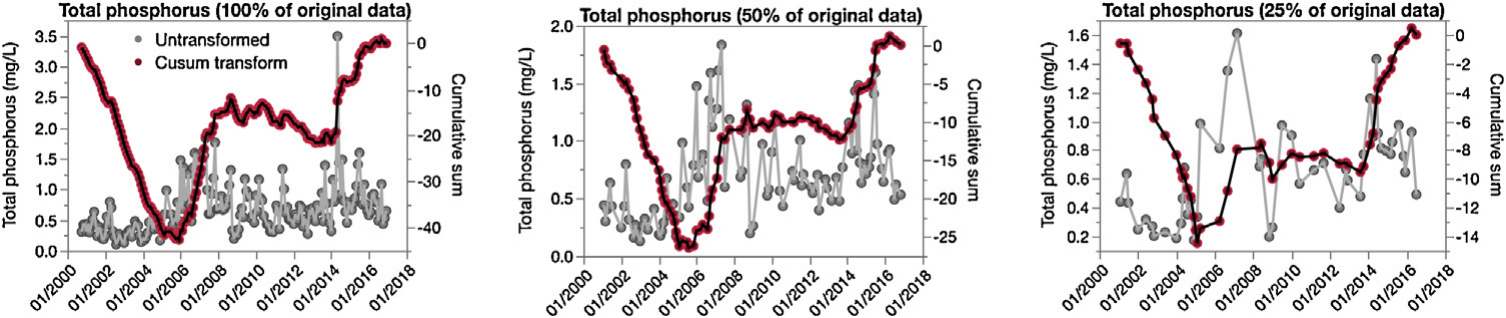
Cumulative sums calculated from the total phosphorus time-series presented in Fig. 1, with data-points randomly removed to demonstrate cumulative sums as a method robust to data gaps. Untransformed time-series are presented in gray, while cumulative sums are presented in red. A) All data and accompanying cumulative sums transformation (0% removed), B) 50% of original data (50% removed), C) 25% of original data (75% removed).

Gaps in the raw time series were synthesized by randomly resampling the dataset to “punch holes” in the data, and then recalculating Cusums (Fig. 2). Even with 75% of the original dataset removed, all three Cusum graphs showed very similar patterns (Fig. 2B and C), with a shift from below average to above average in 2005, a period of near-average values until 2014, and above average values after 2014. Removing half of the data results in a very similar Cusum (Table 1, NSE = 0.968), while removing 75% results in a noticeable difference between the full and gapped datasets (NSE = 0.792). However, this indicates that calculating the Cusums from only 25% of the original data still matches ˜80% of the full Cusum correctly.

**Table 1:** Nash Sutcliffe model efficiency (NSE) against Fig. 1C Cusum.

*Cusum tested*	*Figure*	*NSE*
50% of original data	2B	0.968
25% of original data	2C	0.792
Square root	3A	0.986
Squared	3B	0.946
Log-10	3C	0.945
+100% white noise	4A	0.985
+200% white noise	4B	0.954
+1000% white noise	4C	0.735

**Fig. 3:**
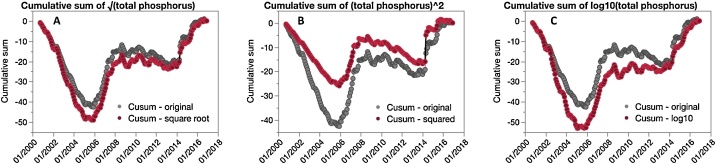
Cumulative sums calculated from the total phosphorus time-series presented in Fig. 1, with data transformed to test sensitivity of cumulative sums transformations to different data distributions. Cumulative sums of transformed data are presented in red, with the cumulative sum of the original data presented in gray for reference. A) Cumulative sum of the square root of original data, B) cumulative sum of the square of the original data, and C) cumulative sum of log-10 transform of the original data.

To test the independence of the Cusum transformation from the distribution of the untransformed dataset, TP data were transformed using square-root, square, and logarithm (log_10_) transformations. These operations are commonly used to transform datasets into normal distributions. After transformation, Cusums were calculated for the original and transformed datasets (Fig. 3). Comparison with original data consistently rendered high NSE values for all three transformations (all greater than 0.945; Table 1). It is important to mention that only the log-10 transformation provided a distribution not significantly different from a normal (Gaussian) distribution (p > 0.05, Shapiro-Wilk Test), while the squared, and square-root transformations were both significantly different (p < 0.01). Our results indicate that changing data distribution does not affect the key characteristics of the TP Cusum curve, and the method appears to be insensitive to the tested transformations.

**Fig. 4:**
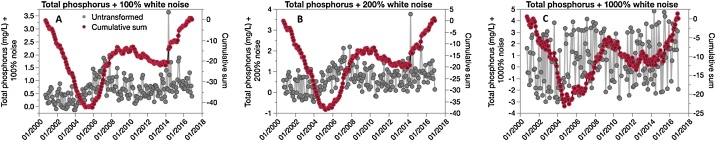
Cumulative sums calculated from the total phosphorus time-series presented in Fig. 1, with addition of white noise to test sensitivity of the cumulative sums transformation to noisy datasets. Cumulative sums of time-series with increased noise are presented in red, with the cumulative sum of the original data presented in gray for reference. A) Cumulative sum of the original time-series with +100% white noise, B) cumulative sum of the original time-series with +200% white noise, and C) cumulative sum of the original time-series with +1000% white noise.

Finally, the impact of noise, a common attribute in environmental time series, was tested by synthesizing white noise and adding to the original TP time series (Fig. 4). A 100% increase in white noise was simulated by randomizing values with a range proportional to two times the dataset mean (0.68 mg L^−1^, range of +100% noise: −0.68 mg L^−1^ to 0.68 mg L^−1^). The mean was selected to set the range of the white noise generated as it was larger than either the dataset median or standard deviation (median: 0.60 mg L^−1^, SD: 0.42 mg L^−1^). Cusums with added noise visually maintain key characteristics, indicating the Cusums method is robust even to high levels of noise (Fig. 4). NSE results in Table 1 confirm that both 100% and 200% additions of noise did not strongly influence predictive power (NSE = 0.985 and 0.954, respectively), although the addition of 1000% noise did lower the NSE to 0.735.

Based on visual comparisons between the original Cusum and Cusums altered with added data gaps (Fig. 2), altered distributions (Fig. 3), and added white noise (Fig. 4), the key characteristics of the Cusum (i.e. the location of breaks and positive/negative slopes) are visually consistent, even under extreme conditions (i.e. removing 75% of the dataset or adding large amounts of noise). Using an NSE threshold of 0.65 established in the literature ([22,23]), none of the confounding factors applied to data significantly affected the outcome of the Cusums transform.

**Fig. 5:**
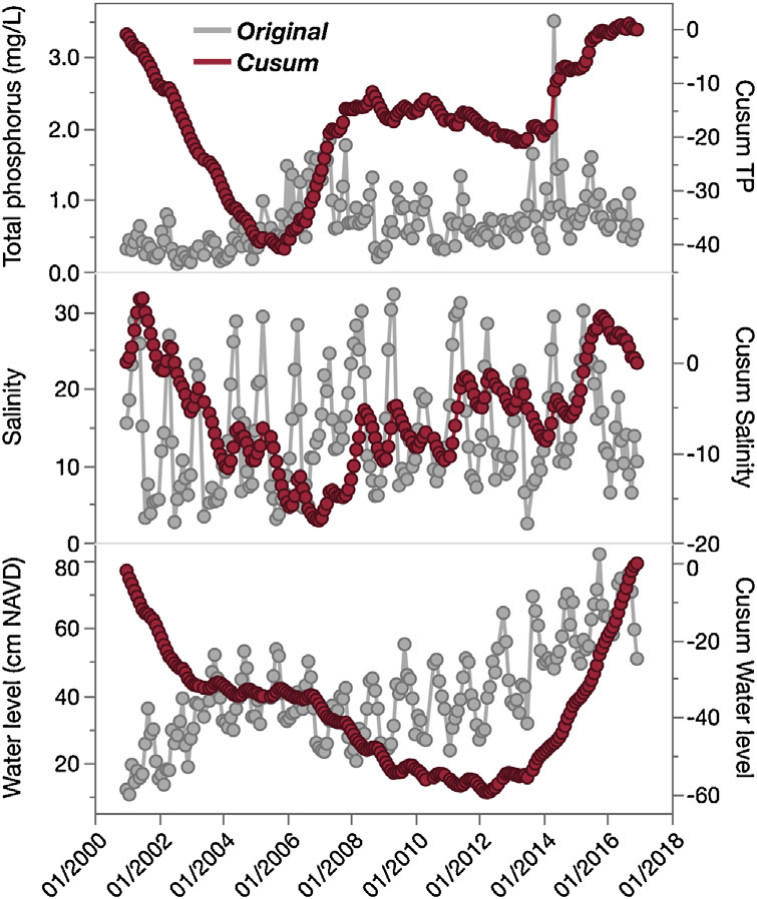
Original (gray) and Cusum-transformed (red) time-series for total phosphorus (TP), salinity, and water level at SRS-5.

To demonstrate the ability of Cusums to quickly and simply explore changes over time in complex environmental time series, data for TP, salinity, and water level at SRS-5 collected from two datasets (LT_ND_Grahl_001, [19]; PHY_Grahl_002 [24]) are presented as original and Cusum-transformed time series, which have been binned to monthly arithmetic means (Fig. 5). Unaltered data exhibit high levels of noise, complex seasonal behavior, and apparent underlying long-term trends. All time series were Cusum-transformed using Eq. (2), with resulting time series presented along with original data. Based on the side-by-side comparison in Fig. 5, it is clear that Cusums provide a simple yet valuable tool to easily interpret initial patterns and trends in complex datasets which are not obvious in the raw data. For instance, increasing trends are immediately identified for all three parameters, given their generalized bowl-like shape in the Cusum-transformed time series in Fig. 5. This bowl-like shape is developed because early in the time series, values were predominantly below-average, rendering a negative slope for the first portion of the Cusum line-plot. Later, values increased above the dataset average, developing a positive-slope curve. Likewise, Cusums provide preliminary information about seasonality and other time series properties. In Fig. 5, amplitude of seasonal variability in the raw salinity data is large and it masks the secular trend. However, the Cusum-transformed salinity time series clearly shows both seasonality and a longer inter-annual increasing trend. The Cusum-transformed salinity signal clearly defines a long-term increasing trend while preserving the seasonal signal and indicating 3- to 4-year sub-cycles. Moreover, the impact of high-frequency noise is greatly reduced, as observed for TP time series.

### Quantification of driver-response relationships

Using the same dataset from the previous section, driver-response relationships were constructed to demonstrate the efficiency of the Cusum method to provide an initial analysis of complex variable-variable relationships. The driver-response plot is constructed using two variables with paired observations, one that is a potential driver (e.g. time, nutrient concentration, water level, etc.), and the other a potential response. First, the paired measurements are ordered so the driver variable is organized in ascending order. The reordered response variable is then Cusum-transformed following Eq. (2).

**Fig. 6:**
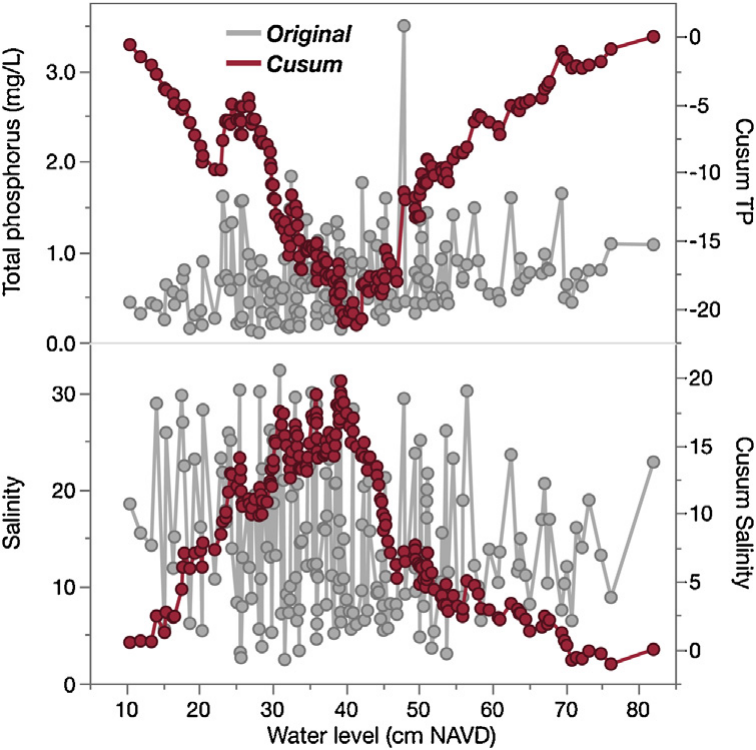
Original (gray) and Cusum-transformed (red) total phosphorus (TP) and salinity data plotted against corresponding untransformed water level measurements.

Water level at the SRS-5 estuarine site represents a combination of hydrologic factors, including increased freshwater inputs (and delivery of associated nutrients) from upstream marshes during the wet season, increased saltwater intrusion during the dry season, and semi-diurnal tidal cycles. To explore water level as a potential driver of TP and salinity (and the benefits of Cusums over untransformed time series in this capacity), the two potential response variables (TP and salinity) were plotted as untransformed and Cusum-transformed formats against untransformed water level (Fig. 6). Untransformed data provide little to no information regarding relationships between response variables and water level, in spite of the expectation that water chemistry parameters will reflect changes in water level given the shifting hydrology and nutrient sources. However, Cusum-transformed TP and salinity show clear relationships to water level, where TP increases with higher water level (bowl-shaped Cusum), and salinity decreases (dome-shaped Cusum).

### Use of Cusums in threshold analysis

The change points where Cusum curves reverse slopes in Fig. 6 (negative to positive for TP, positive to negative for salinity) provide further useful information. TP concentrations shift from below average to above average around 40 cm water depth, while the opposite is true for salinity. Such simple and quick analysis of driver-response relationships in the complex time series highlight the value of Cusum charts to easily examine underlying relationships, which cannot be directly observed in untransformed time series. The authors anticipate that thresholds established in driver-response Cusum charts will be a useful tool for ecosystem management, as discussed by Andersen et al. [1] and others. For instance, Fig. 6 suggests that water levels below ˜40 cm at SRS-5 correspond to lower TP concentrations. Long-term research in the Shark River conducted by the FCE-LTER program indicates an “upside-down estuary” phenomenon, where phosphorus (the limiting nutrient in this system) is sourced from the marine endmember rather than freshwater inflows [25]. As Everglades restoration efforts focus on altering freshwater flows upstream of Shark River Slough to manage phosphorus [26], this relationship could be used to guide desired water levels to manage the level of phosphorus in the estuary. In this way, Cusums may provide a useful management tool to establish guidelines for controlling one parameter based on historic relationships to another (in this case, TP and water level respectively). Moreover, such driver-response relationships may be useful when applied to readily available data (i.e. water level) to estimate parameters which may be measured less frequently or are more expensive to analyze (i.e. TP).

### Limitations to Cusum-transformed time series

It is important to note limitations to the format of data compatible with the Cusum techniques described above, particularly irregular time intervals between sampling events. Since neither Eq. (1) or Eq. (2) incorporates the time interval between data points, regular sampling intervals are assumed. However, irregular sampling frequencies due to any number of potential limitations (e.g. seasonal lack of access to site, ice-over of aquatic sites, etc.) are common in environmental studies. This issue is illustrated in Fig. 2, where holes were randomly punched in the time series. The authors do observe some deformation of the Cusum shape, although NSE statistics in Table 1 indicate minimal change when half the data are randomly removed, and, even with 75% of the data randomly removed, the resulting Cusum is still well predicted (based on the previously discussed NSE threshold of 0.65 in the literature).

It has also been documented that Cusum-transformed time series are not appropriate for linear regression, where the Cusum transformation increases auto-correlation in a time series, violating linear regression assumptions that data are independently sampled and identically distributed [27]. As such, applying linear regression modeling to two Cusum-transformed time series can result in spuriously high correlations. In the two examples of Cusum plot types presented in this manuscript, the variable on the y-axis is Cusum-transformed, while the variable on the x-axis is not, and we are not applying linear regression analysis. However, the authors caution that users of the Cusums methodology should be aware of potential limitations discussed in this section before using the technique.

## Conclusions

Cumulative sums represent a valuable statistical tool with broad interdisciplinary applications to interpret patterns in complex time series without the need to pre-process data. Here, details are presented to simply and easily calculate Cusums and produce two types of plots which are highly useful in understanding behavior of environmental data over time, and in relation to potential driver variables. Both plots are applied to environmental datasets that initially suffer from high signal-to-noise ratios, strong seasonality, and few obvious tendencies. Through application of the Cusum plots described with minimal pre-processing, meaningful information can be easily extracted from these complex signals. There is currently an abundance of publicly available long-term environmental datasets which have not been interpreted due to the time and cost required to process and analyze them. As such, authors believe the Cusum method, which is easily learned and requires no special software other than an ordinary spreadsheet application (see our example spreadsheet in supplementary material), is an ideal tool to assist environmental scientists in more efficiently inspecting and gaining insight from long-term environmental data, before applying more sophisticated statistical algorithms.
